# Distributed Fiber Optic Sensing for Real-Time Monitoring of Gas in Riser during Offshore Drilling

**DOI:** 10.3390/s20010267

**Published:** 2020-01-02

**Authors:** Giuseppe Feo, Jyotsna Sharma, Dmitry Kortukov, Wesley Williams, Toba Ogunsanwo

**Affiliations:** 1 Department of Petroleum Engineering, Louisiana State University, Baton Rouge, LA 70803, USA; gfeo1@lsu.edu (G.F.); wcwilliams@lsu.edu (W.W.); 2 Schlumberger, Sugarland, TX 77478, USA; DKortukov@slb.com; 3 Schlumberger Canada, Calgary, AB T2G 0P6, Canada; OOgunsanwo@slb.com

**Keywords:** distributed fiber optic sensing, offshore drilling, gas kick

## Abstract

Effective well control depends on the drilling teams’ knowledge of wellbore flow dynamics and their ability to predict and control influx. Unfortunately, detection of a gas influx in an offshore environment is particularly challenging, and there are no existing datasets that have been verified and validated for gas kick migration at full-scale annular conditions. This study bridges this gap and presents pioneering research in the application of fiber optic sensing for monitoring gas in riser. The proposed sensing paradigm was validated through well-scale experiments conducted at Petroleum Engineering Research & Technology Transfer lab (PERTT) facility at Louisiana State University (LSU), simulating an offshore marine riser environment with its larger than average annular space and mud circulation capability. The experimental setup instrumented with distributed fiber optic sensors and pressure/temperature gauges provides a physical model to study the dynamic gas migration in full-scale annular conditions. Current kick detection methods primarily utilize surface measurements and do not always reliably detect a gas influx. The proposed application of distributed fiber optic sensing overcomes this key limitation of conventional kick detection methods, by providing real-time distributed downhole data for accurate and reliable monitoring. The two-phase flow experiments conducted in this research provide critical insights for understanding the flow dynamics in offshore drilling riser conditions, and the results provide an indication of how quickly gas can migrate in a marine riser scenario, warranting further investigation for the sake of effective well control.

## 1. Introduction

Recent developments in offshore drilling technology and automation have made the mobile offshore drilling unit an engineering marvel. In the face of these developments however, kick detection, which is relatively straightforward on land based units, has proven significantly more challenging to master on a marine vessel subject to currents and wave motion. A gas kick is a well control problem that results from an influx of gas (or kick) into the wellbore during drilling operation. It occurs when the pressure in the wellbore is lower than that of the formation fluids, thus causing flow. Factors such as inadequate equivalent circulating density (ECD), higher than expected formation pressures, mud losses during drilling, and swabbing can all trigger a gas kick [1]. Usually, the influx is removed by circulating the well by means of an adjustable surface choke [2].

A primary challenge for deepwater well control is the increase of water depth causing the safe mud window to gradually become narrower, making the possibility of a gas kick more likely. Secondly, due to high pressure environment at high water depths, the overall scale of a gas kick is relatively small and the increment of the mud pool may not reach the warning value when the gas bubbles reach the subsea wellhead [3]. In other words, the gas at the bottom of the riser under high hydrostatic pressure occupies a relatively small volume, making it difficult to cause an increase in pit gain at the surface. This condition is exacerbated at the shallow depths, such as in the Gulf of Mexico gas sands. Additionally, if the formations are relatively thick or tilted, they can potentially require significantly higher mud weights even though they are not abnormally pressured [4].

With the increasing use of longer risers with greater volume and weight, there still remains a general lack of consensus regarding suitable standards and regulations regarding kick detection. The API 53 standard [5] of a trip tank, a Pressure Volume Temperature (PVT) system, and a return flow sensor is still based on conventional kick detection philosophy and lacks specificity to properly mitigate gas in riser scenarios. As deepwater and ultra-deepwater drilling continue exploring for the most productive plays in increasingly challenging offshore environments, a recipe is formed such that a slight variation between formation pressure and wellbore pressure has the potential to draw a substantial gas volume into the system [6]. Hence, the study of kick detection is fundamental to the safe and reliable offshore hydrocarbon production. The next few sections summarize the key limitations of conventional kick detection techniques, and present a novel application of distributed fiber optic sensing technology for real-time distributed downhole monitoring—unachievable using traditional methods.

### Limitations of Conventional Kick Detection Methods 

Conventional well control procedures primarily utilize a series of surface measurements, and even though they are somewhat reliable, they consume valuable response time and potentially aggravate the initial problem of the gas influx [6,7,8,9]. For example, stopping circulation and shutting in a well decreases the ECD in real time, and although this is standard operation for killing the well, it reduces the hydrostatic pressure and aggravates the drawdown of a gas influx already occurring. The four main surface methods to detect kicks and their main limitations are described in Table 1 [6,7,8,9,10,11,12,13]. The limitations of the conventional kick detection methods, combined with the narrow drilling windows typical of deepwater conditions, make accurate and timely kick detection challenging.

## 2. Fiber Optic Sensing as an Early Kick Detection Method

Fiber optic sensing is a promising real-time downhole sensing technology for early kick detection since it can be deployed on the marine riser at working conditions with minimal interference with system performance and dimensions. Given the demands for chemical and electrical passivity, transmission lengths, and operational robustness, fiber-optic sensing methods are an excellent way of monitoring marine riser systems [13]. The methods of fiber optic sensing can be adapted for use in the riser environment, providing a means of real-time measurement with high spatial resolution along the length of the fiber itself. Fiber optic surveillance overcomes the key limitation of conventional kick detection methods—which primarily rely on surface measurements at a limited set of locations (point sensing)—by proving direct assessment of downhole conditions along multiple locations (distributed sensing). Thus this emerging technology has the potential to significantly improve our ability to detect and manage gas kick and ensure safe offshore drilling.

### 2.1. Overview of Distributed Fiber Optic Sensing Technology

**Figure 1:**
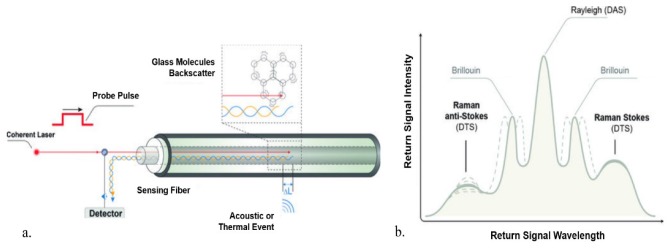
(**a**) Schematic of distributed fiber optic sensing. (**b**) Backscattered signal [14].

A typical set up for distributed fiber optic sensing is shown in Figure 1a [14]. An intense laser pulse is launched into the sensing fiber yielding spontaneous scattering as it interacts with the crystalline structure in the silica-based core of a fiber optic cable. The glass structure is affected by effects such as thermal and pressure variations (noise/sound) causing oscillations in the glass core. A fraction of the scattered photons are captured in the guided modes of the fiber and propagated back towards the interrogator where they are detected by a fast photodetector [15]. The scattering is caused by the interaction of the laser light with density fluctuations within the fiber. The light’s backscattered spectrum consists of a Rayleigh band, Brillouin band, and Raman band, as shown in Figure 1b [14]. The main fiber optic measurements widely used today are the Distributed Temperature Sensing (DTS), Distributed Acoustic Sensing (DAS) and Distributed Strain Sensing (DSS) [16]. Where, DAS is measured from Raleigh scattering, DTS from Raman scattering, and DSS from Brillouin scattering. Thus, the changes in the back-scattered light can be related to the acoustic and thermal variations along the fiber [17]. Distributed sensors function via optical time domain reflectometry (OTDR), using the technique of location of a local loss in the fiber. Knowledge of the propagation time of a pulse at a particular wavelength along a fiber of specific refractive index enables the position of the interaction to be located and, when used for sensing, the measurand perturbation on the fiber to be determined.

Fiber optic monitoring implemented in downhole conditions provides signals of fluid flow along the wellbore in addition to potentially serving as a sensor for wellbore seismic readings, thermal imaging, and completion efficiency. There are three main methods for deploying fiber optics down the wellbore: permanent installations, semi-permanent installations and temporary or retrievable installations. A permanent installation of fiber optics involves cementing the fiber behind the casing. A semi-permanent installation can be implemented by clamping the fiber optic line to the tubing, such as the one performed on the PERTTtest well (described in Section 2). This enables the capability to retrofit existing wells with fiber optics. The two aforementioned installation methods require a higher capital cost and lower operational cost, but their main advantage lies within their capability to instantly acquire downhole measurements without having to access the wellbore simply by attaching either the DTS or DAS interrogator, or both, to the fiber optic cable easily available at the surface. The temporary or retrievable installation method is deployed via fiber optic embedded wireline/slickline [17]. There are numerous field examples of successful deployment of DTS/DAS for injection and production flow profiling, cross-flow determination, flow behind casing, etc. [17,18,19,20].

### 2.2. Fiber Installation and Surveillance on a Marine Riser

To enable real time surveillance on a marine riser using distributed fiber optic sensing, special installation procedure will be implemented. To date, the industry has the experience in the installation of downhole optical cables as well as flexible production riser.

**Figure 2:**
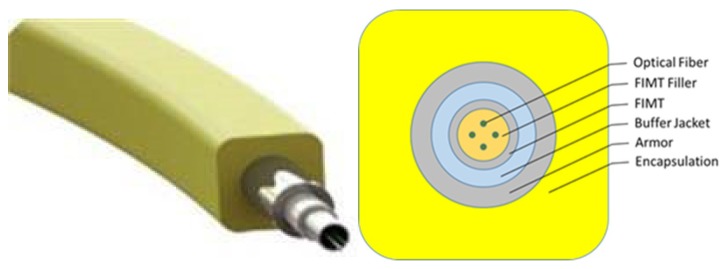
Permanent optical cable design and components [21].

Downhole permanent optical cable comprises of optical fiber, filler, fiber in metal tube (FIMT), a buffer jacket, armor, and encapsulation, as shown in Figure 2. The permanent optical cable is made with controlled overstaffing, which is the manufacturing process to prevent strain and fiber-breaking. It is also created in a moisture environment to manage tension and maximize the hydrogen resistance of the fiber. These factors protect the enclosed optical fiber from pressure, humidity, expansion, bending, and hydrogen attack.

**Figure 3:**
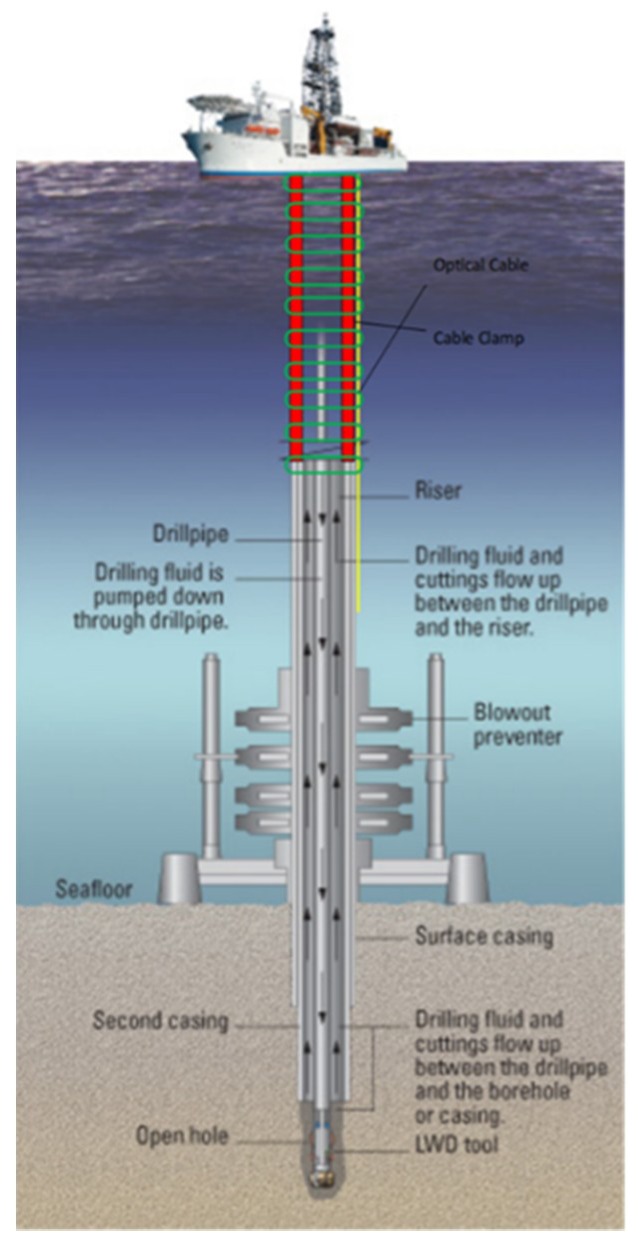
Schematic of optical cable installation on a flexible production riser in an offshore well (modified from [21]).

Standard way to install such cable is to strap it to deployment tubing/casing using metallic clamps. They are installed on every joint connection to ensure distributed mechanical support for the cable. For flexible production riser monitoring, such cable can be installed on one of metal rods, forming the umbilical, as illustrated in Figure 3. Since the riser is a large-diameter pipe that connects the subsea blowout preventer (BOP) stack to a floating surface rig to take mud returns to the surface, it can be loosely considered a temporary extension of the wellbore to the surface. Therefore, it will be similar in terms of mechanical integration with a downhole optical cable.

Such approach will ensure robustness and reliability of installation, while maintaining high quality of optical measurements. Cable design will ensure mechanical protection of the fiber and resistance to corrosion. Upon installation the cable will be secured to riser, using special cable clamps. They will ensure that the cable stays in place, as well as mechanical coupling of it to the riser. Such mechanical coupling improves quality of transfer of temperature and acoustic events from within the riser to the cable through the riser wall. The bottom end of the cable will be protected with special sealing mechanism, which may include fiber loop, which provides higher quality of the signal, but also provides robust sealing against water ingress [22]. The top part of the cable will be terminated at rig level via a special Fiber Termination Box. This Termination Box provides sealing of both cable mechanical protecting layers as well as the fiber itself. From the Termination Box, another surface optical cable will perform optical connection and extend it all the way to the room with an Interrogator Unit.

In such deployment strategy there is confidence in the fact that acoustic signals from gas slugs travelling upwards will be sufficiently transferred through the riser wall into the sensing cable, so that it can be distinguished from other environmental noise. For the temperature anomalies, however additional thermal isolation may be required and is the subject for additional analysis.

## 3. Materials and Methods

**Figure 4:**
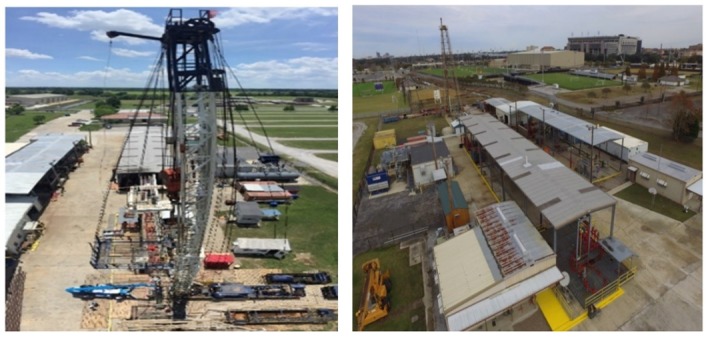
Petroleum Engineering Research & Technology Transfer (PERTT) lab facility at LSU.

The downhole distributed sensor involving fiber optic DTS and DAS were performed at the PERTT lab facility at LSU, shown in Figure 4. PERTT lab is a world-renowned facility for the development, integration and testing of technologies used in the oil and gas industry. There are six wells, up to 5800 ft in depth that provide a unique full-scale environment to test downhole equipment on field scale tubulars at high pressures. PERTT currently has underground storage volume in three wells that can supply up to 180,000 standard cubic feet of gas, pressurized up to 4500 psi. An existing 6 in natural gas line gives access to gas at 550 psi in large volumes that can be compressed into the storage wells with a Norwalk Compressor. The tubulars in the well will allow for the simulation of drilling circulation with flow rates up to 600 gallons per minute and maximum gas kick volumes up to 25 barrels. The facility has the ability to inject high pressured gas at 4000 psi and up to 20 MMSCFD, which is unique for a controlled experimental wellbore. It also has the flexibility to set tools through wireline/slickline as well as the ability to circulate multiphase fluids at full scale.

### 3.1. Test Well Description

**Figure 5:**
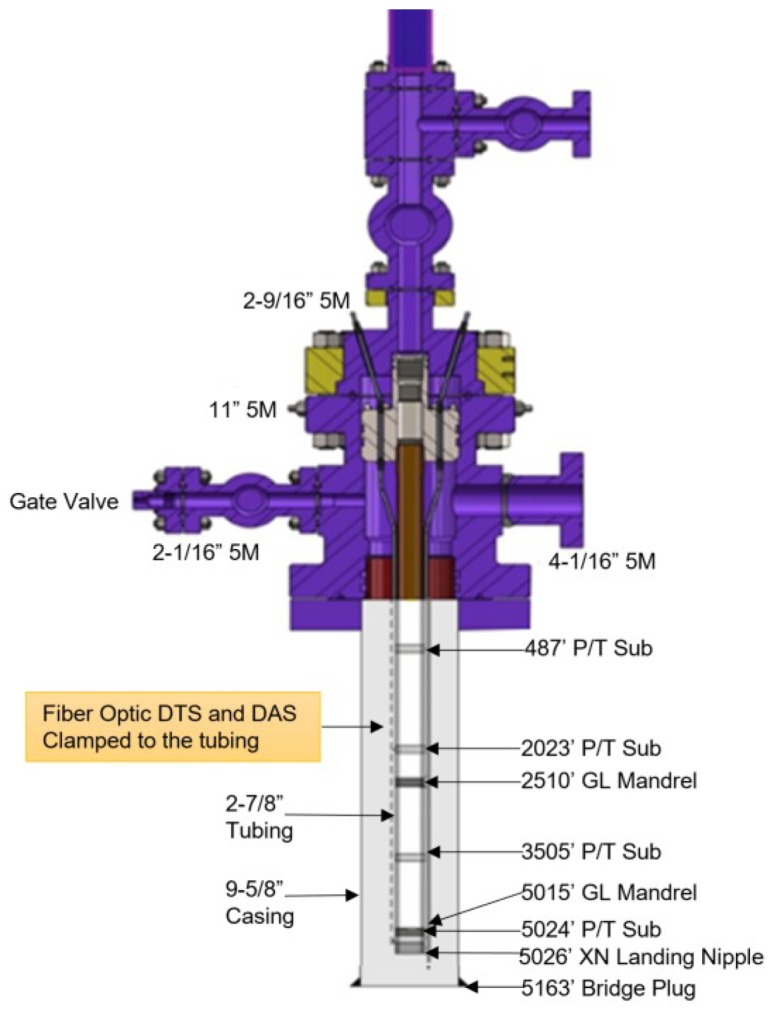
Test well schematic.

The test well is a 5800 ft deep, 9-5/8 in cased and cemented wellbore with a bridge plug at 5163 ft, as shown in the well schematic in Figure 5. Currently the wellbore has a 2-7/8 in production tubing up to 5007 ft, with a landing nipple at the bottom, exactly like what would be found in a producing oil and gas well. The casing is 17 lb/ft, J-55 pipe. Initially the test well is full of water. Nitrogen was injected down the tubing to simulate gas influx in the well. An existing choke and orifice meter was used for measurement and control of injected volumes. The larger than normal annular volume was used to simulate gas rise in a marine riser-like scenario.

### 3.2. Workover and Fiber Installation

**Figure 6:**
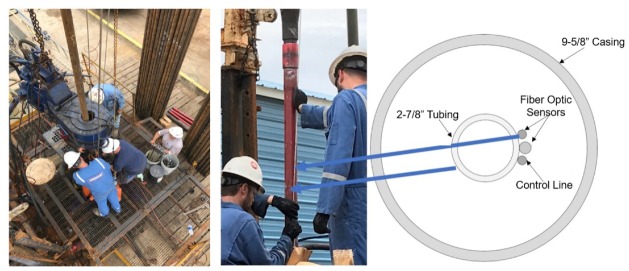
Test well workover and fiber installation performed by Schlumberger.

The test well was retrofitted with DTS and DAS, as well as four downhole pressure and temperature gauges (marked as “P/T sub” in Figure 3), over a 5000 ft vertical interval in a recent workover in the PERTT test well. Table 2 summarizes the surveillance parameters for the DTS and DAS units installed on the test well. The fiber installation and workover is shown in Figure 6. The first workover task was to pull the existing tubing out of the well, so that new tubing could be installed. Once the directional and casing integrity surveys were completed, a bridge plug was set at 5163 ft. The workover rig proceeded to lower the new tubing down the hole while simultaneously instrumenting it. The fiber optic cable was secured to the outside of the 2-7/8 in L-80 6.5# tubing using bands about 6–8 ft above and below each joint, and each tubing was pressure tested before being deployed downhole. Each pressure gauge was also pressure tested before deployment, and additional time was needed for each gauge to make connections with the electric line. In addition to the fiber optic line and electric line for the gauges, two gas lift mandrels (marked as “GL Mandrel” in Figure 5) and a chemical injection line was also installed up to 5015 ft. Once the 5000 ft of instrumented tubing was downhole, a simple OTDR signal was employed to ensure the functionality of the fiber optic cable, and the pressure gauges were also verified to be functional producing real-time measurements on display.

### 3.3. Methodology

The experiment was conducted in three stages to capture two-phase flow effects in static and circulating conditions, to mimic gas in riser scenario, as described below.

- **Stage-0, Baseline** [Duration: 19:25–19:27]: Baseline data for hydrostatic pressure and geothermal gradients was obtained in a static fluid column.
- **Stage-1, Gas Injection** [Duration: 19:28–19:39]: Nitrogen gas was injected down the tubing at 110 psi from a nearby storage well, with the gate valve in the closed position to prevent any outflow.
- **Stage-2, Water Circulation** [Duration: 19:40–20:35]: Water was injected down the tubing at a pump rate of 4 bbl/min pushing gas downwards and eventually upwards through the annulus. The gate valve was opened to initiate circulation through the annulus at 19:43.
- **Stage-3, No Circulation** [Duration: 20:36–21:31]: Water circulation was stopped and free movement of injected gas was observed.

## 4. Results and Discussion

This section describes the results from the DTS, DAS, and pressure/temperature gauges obtained during the different stages of the experiment, described in the previous section.

**Figure 7:**
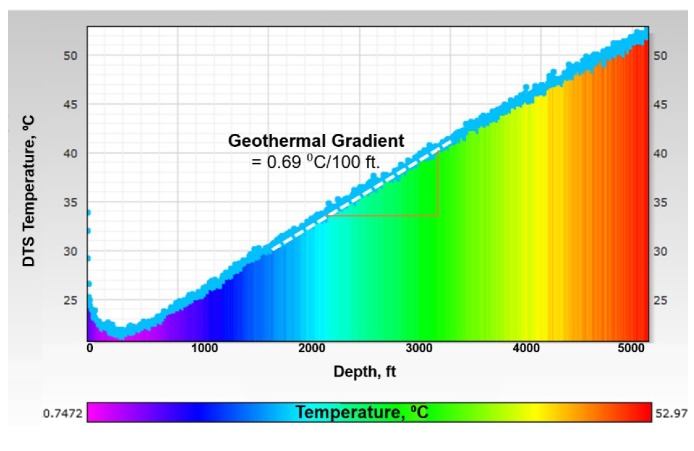
Baseline temperature (Stage-0) showing geothermal gradient.

**Figure 8:**
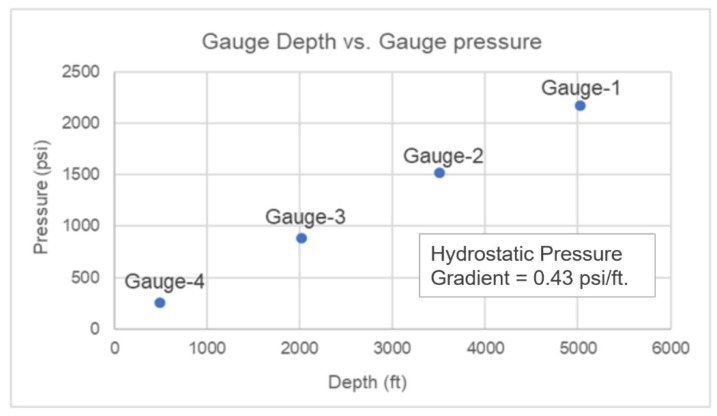
Hydrostatic pressure gradient (Stage-0).

**Stage-0, Baseline**: Baseline data was established for the pressure and temperature in a static water column inside the test well. Figure 7 shows the geothermal temperature profile, with a temperature gradient of about 0.689 °C/100 ft (or 1.24 °F/100 ft). Figure 8 shows the hydrostatic pressure profile with a gradient of about 0.43 psi/ft.

**Figure 9:**
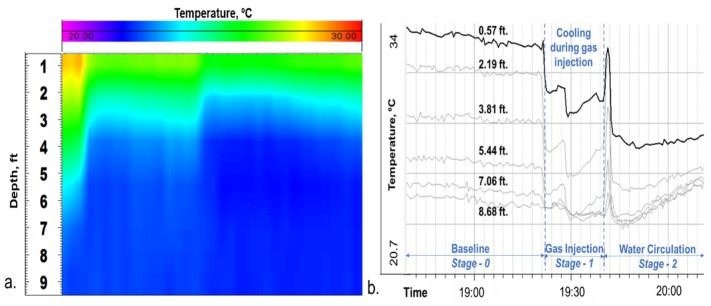
Temperature drop at shallow depth due to gas injection during Stage-1. (**a**) DTS waterfall. (**b**) Temperature traces at six different depths showing the temperature drop reducing at higher depths.

**Figure 10:**
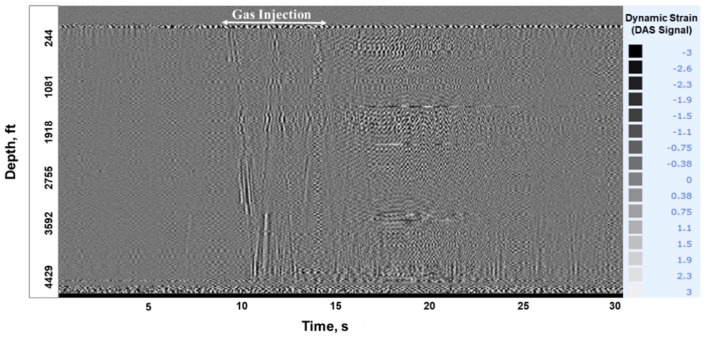
Acoustic signal created from gas injection in Stage-1 is visible in a 30 s. DAS segy trace, taken at 19:28.

**Stage-1, Gas Injection**: At 19:28, compressed Nitrogen gas at 110 psi was injected down the tubing. Figure 9a shows the DTS waterfall chart displaying a temperature drop close to the surface upon gas injection. Figure 9b shows the corresponding temperature traces as the gas travels down the tubing, seen at six depths close to the surface. These figures illustrate a temperature drop upon gas injection in a static water column, which is a result of gas expansion and the relatively cooler temperature of injected gas compared to the water in the tubing. The temperature drop is more prominent at shallower depths as the gas enters the tubing, but it quickly dissipates at deeper intervals (greater than 9 ft) as the gas continues to compress in the tubing column. Acoustic signal created by gas injection is also clearly visible in the raw DAS data (in segy format), as shown in a 30 s trace taken at 19:28 in Figure 10. The acoustic effect dissipates towards the end of the injection period. This is because of the gas compression at the top of the tubing column, since the annular gate valve was kept in the closed position to prevent any fluid outflow from the annulus.

**Figure 11:**
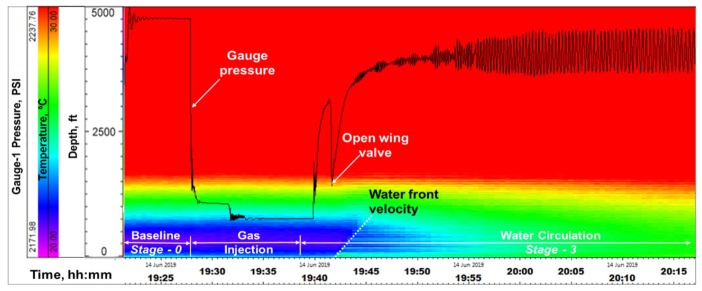
Water front movement seen in DTS in Stage-2.

**Figure 12:**
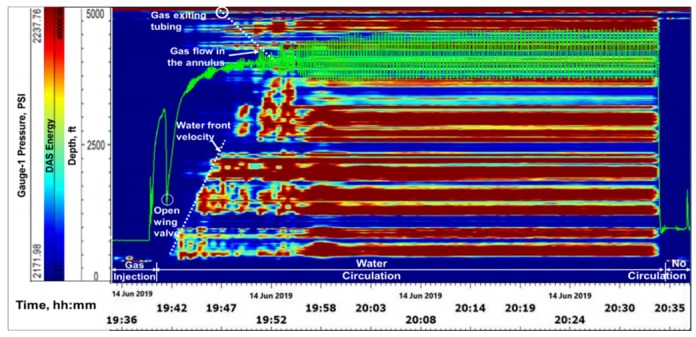
Water front movement seen in DAS during water circulation in Stage-2.

**Figure 13:**
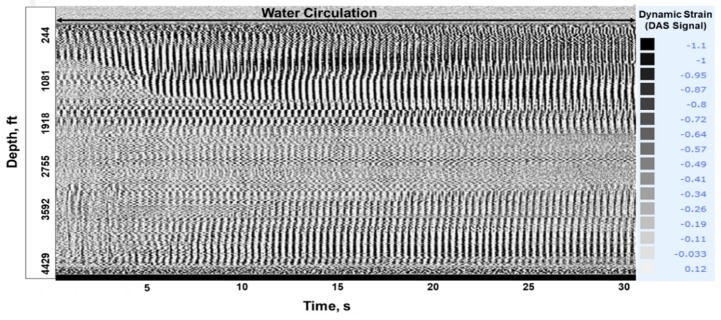
Acoustic signal created from water circulation after the gate valve is opened is visible in a 30 s. DAS segy trace, taken at 19:43 during Stage-2.

**Stage-2, Water Circulation**: At 19:40, gas injection was stopped and water circulation was started at a rate of 4 bbl/min down the tubing. The objective here was to see the movement of the gas slug in a circulating fluid column, to mimic riser condition. At 19:43, the annular gate valve (shown in Figure 5, above the wellhead) was opened, relieving the annular backpressure and signaling the initiation of outflow from the annulus, which is seen in both DTS and DAS. Figure 11 shows the water front movement in the DTS waterfall plot. The front velocity was calculated from the slope of the temperature front as ~4.3 ft/s, which corresponds to about 1.5 bbl/min flow rate for a 2-7/8 in tubing. Water front movement is also seen in the DAS waterfall plot in Figure 12, which shows a sum of all frequency band signals or Band-0 corresponding to 0–5000 Hz. The gauge pressure (from gauge-1) is overlaid for reference in Figure 11 and Figure 12. The water front velocity down the tubing was calculated from the slope of the acoustic energy propagation in time, shown in the DAS waterfall (Figure 12), to be roughly 5.3 ft/s, or 1.85 bbl/min. This is higher than the water front velocity calculated from the DTS traces, as thermal propagation is typically slower than acoustic propagation due to thermal inertia, which is a function of the heat conductivity and capacity. Beginning of water movement as gate valve is opened is also seen in the raw DAS segy trace taken at 19:43, shown in Figure 13.

**Figure 14:**
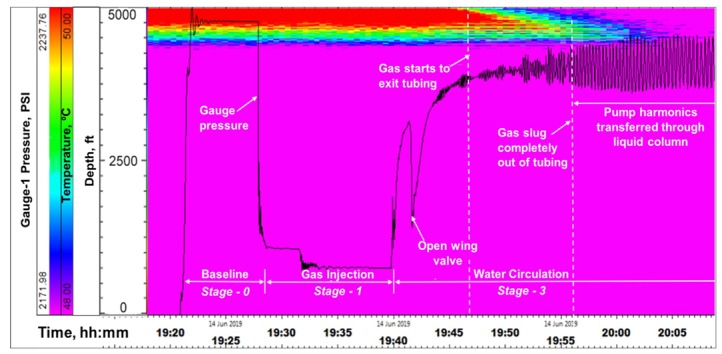
Arrival of gas at the end of tubing and complete exit into the annulus is seen in DTS.

As the water continued to be injected, the first arrival of gas in the annulus after going down the entirety of the tubing can be seen in DAS and DTS, in Figure 12 and Figure 14, respectively. DTS waterfall plot in Figure 14 shows the arrival of gas at around 19:46 corresponding to a decrease in temperature near the bottom of the tubing. This is due to the adiabatic effect of the gas expanding from a small tubing volume to a relatively large annulus volume. Gas arrival at the end of tubing and propagation in the annulus is also observed in the DAS waterfall plot in Figure 12, around 19:46, confirming the interpretation. It is believed that during this time the gas bubbles are dispersed in the water, and hence the acoustic front in the annulus is not sharp. Higher oscillations in the pressure gauge data seen in Figure 12 and Figure 14, also indicates gas completely exiting the tubing (around 19:56), as the pump harmonics are transferred more rapidly through the liquid-filled column in the tubing, after gas exits the tubing. The gas front velocity from the slope of the acoustic propagation was calculated to be about 2–3.2 ft/s, or 0.8–1.2 bbl/min. While this is an approximate value, given the dispersed flow regime, an estimate of the gas rise rate is nonetheless an important parameter for well control strategy in a gas in riser situation. The estimated slopes of both the water and gas fronts from the DAS data yielding 1.85 bbl/min and 1.15 bbl/min, respectively, correspond to a total flowrate of around 4 bbl/min, which was the actual pump rate, thus substantiating these measurements.

It is widely believed in the drilling industry that gas rises through drilling mud at 1000 ft/h or 0.27 ft/s [23], however the experiments conducted for this work are indicating a higher rising gas velocity of 3.2 ft/s. This is because gas-rise velocity has been proven to increase when in an annular environment compared to a pipe environment, and also increase as the area of the annulus increases [2]. The circulating conditions during the experiment also enabled a faster rise rate. Previous experimental work [24,25] on a 12 m 7.8 in. ID tubing resulted in maximum gas velocities of only 0.2 ft/s. Moreover, analysis of gas rise velocities at an experimental well facility in Norway, in a 2020 m long research well simulating a gas kick, concluded that the gas rise velocity was around 0.27 ft/s. However, in their experiment, the gas was injected inside the drilling string through a coiled tubing and not the annulus, so the results of this experiment yielding a high-end gas-rise velocity of 3.2 ft/s highlight both the significance of circulating conditions and full scale annular geometry, which both cause an increase in gas rise velocity. In effect, this work bridges the gap of previous work failing to include the full-scale circulation in annular environment, for which our preliminary experimental data is indicating a much higher gas rise velocity.

**Figure 15:**
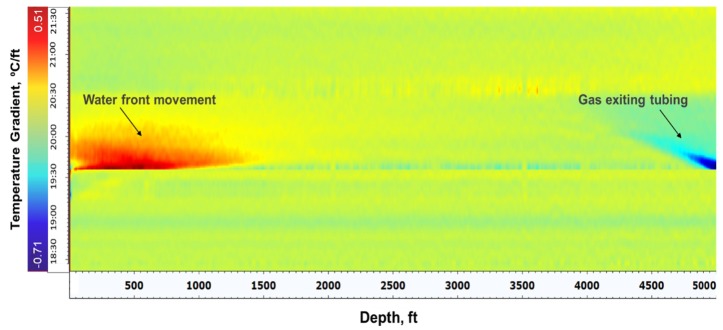
Temperature gradient with respect to time shows water front movement at the top and gas exiting near the bottom of the tubing.

The waterfall plot of temperature gradient with respect to time in Figure 15 also shows the water front movement at the top of the tubing, where the temperature is increasing (positive gradient) as the relatively warm water is injected into the tubing. At the bottom of the tubing, the temperature is decreasing (negative temperature gradient) as the gas is exiting the tubing and cooling due to expansion.

**Figure 16:**
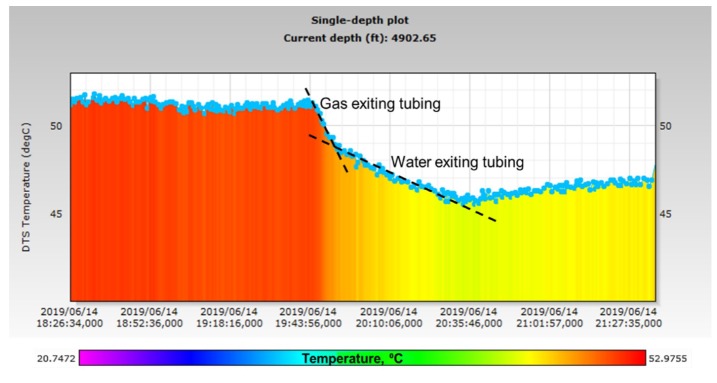
Temperature variation at the bottom of the well shows gas and water exit during Stage-2.

Gas exit from the tubing into the annulus is also established from the single depth plot of temperature variation at the bottom of the well (~4900 ft.) shown in Figure 16. There are two characteristic slopes for the rate of cooling during the two-phase flow of air and water, compared to the rate of cooling for the single-phase flow of just water. The rate of cooling derived from the slope in Figure 16 is 0.5 °C/min during the gas–water phase and a much lower 0.15 °C/min rate of cooling is observed for the single-phase water flow. This is expected as gas cools rapidly due to adiabatic expansion upon entering the large annular space from a relatively smaller tubing.

**Figure 17:**
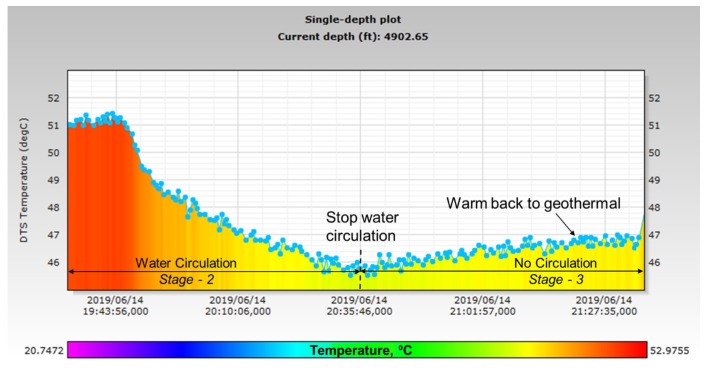
Temperature warm back effect after water circulation is stopped in Stage-3.

**Stage-3, No Circulation**: In this stage of the experiment, water circulation was completely stopped to observe the warm back effect. Figure 17 shows the DTS profile close to the bottom of the tubing (at 4902 ft) during water circulation and after stopping circulation. During water circulation, it is observed that the temperature continues to drop as the injected water displaces the original water at the bottom of the tubing, which is relatively warmer than the injected water due to the geothermal gradient. When circulation is stopped, the temperature begins to warm back to the original geothermal gradient, as seen in Figure 17. The DAS profile during this stage can be seen from Figure 10 (after 20:35), which shows a significant drop in acoustic energy.

**Figure 18:**
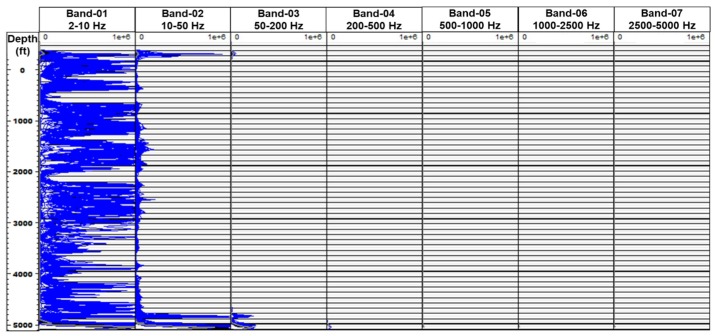
Spectrogram of frequency bands extracted from the raw DAS data shows that majority of the acoustic energy resides in the lower frequency range.

**Figure 19:**
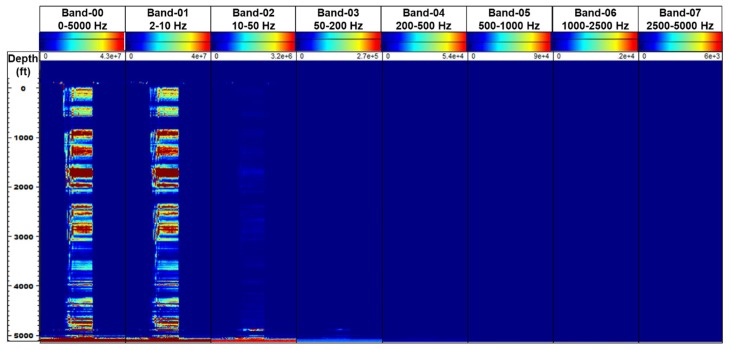
Frequency bands extracted (FBE) signal from the DAS data shows majority of acoustic signal in low frequency bands.

The spectrogram of the frequency bands extracted (FBE) from the raw DAS data (in segy format) shows that the majority of the acoustic energy, resides in the lower frequencies, as shown in Figure 18. Band-1 corresponding to 2–10 Hz frequency range, shows strong signal throughout the entirety of the two-phase flow experiment, while limited features are observed in Band-2 (10–50 Hz) and Band-3 (50–200 Hz), as shown in Figure 19. This is expected due to the low injection rates in the experiments. Here, Band-0 corresponds to the sum total of all frequency bands (0–5000 Hz).

## 5. Conclusions

This paper challenges the concepts of conventional gas kick-detection approach by implementing a real time distributed fiber optic sensing system. The newly instrumented experimental well at LSU’s PERTT facility simulates an offshore marine riser environment with its larger than average annular space and fluid circulation capability at high pressures and rates. Two-phase flow experiments conducted in this study not only substantiate the viability of distributed fiber optic sensing (DTS and DAS) as an early kick detection method, but also present insight into the gas-rise velocity and two-phase flow behavior in a field-scale scenario. The preliminary results from this study, which is the first simulating field-scale annular experiments, shows that gas rise velocity increases substantially because of the large annular conditions as well as circulation, and this warrants further investigation in order to advance our understanding of well control. In addition, the experiments show the capability to track gas movement inside the tubing, as well as in the annulus, using the DTS, DAS, and pressure data which independently corroborate one another. Gas and water front was observed in real-time, both qualitatively and quantitatively, in the raw DAS data as well as frequency band extracted signal, in addition to both temperature and temperature-gradients obtained from DTS.

This preliminary fiber optic data from the two-phase flow experiments in our well-scale facility elucidates the novel application of DTS and DAS for early gas kick detection in deepwater environments. This emerging technology has the potential to significantly improve our ability to detect gas kick as compared to traditional methods, by providing real-time distributed downhole data. Future experimentation will consist of using live circulating drilling muds, where the gas-rise velocity is expected to be affected by the drilling fluid’s non-Newtonian nature.

## Figures and Tables

**Table 1:** Conventional gas kick detection methods and their limitations.

Kick-Detection Method	Description	Limitations
Mud Pit Volumetrics	Measuring volumetric changes in the circulated mud system to detect gas influx.	- Gas influx may not always result in surface volume change. - Requires large surface volume change to reliably detect downhole influx.
Differential Flow	Comparing flow rate in and out of the well to estimate gains or losses of fluid inside the well.	- Requires precise inlet and outlet flow measurements, which is often difficult to achieve using conventional flow meters.
Pressure Variation	Monitoring the pressure at the inlet and outlet (or annular discharge pressure) to detect gas influx.	- Assumes steady-state flow conditions. - Only works with no pipe movement
Flow Modeling	Simulating wellbore flow using hydraulic model and comparing the projected flow rate with actual.	- Difficult to accurately model transient flow and non-linear variations in density rheology, gel strength, etc. in the wellbore.

**Table 2:** Measurement specifications for the fiber optic Distributed Temperature Sensing (DTS) and Distributed Acoustic Sensing (DAS) systems installed on test well.

Measurement Specifications for Fiber Optic Sensors			
DTS		DAS	
Range: Resolution: Accuracy: Spatial Resolution: Sampling Interval: Recording Length: Max Optical Loss:	4 km (2.5 mi) +/−0.1 °C (0.2 °F) 1 °C 1 m (3.28 ft) 0.5–2.0 m (1.64–6.56 ft) 30 s 12 dB (ref. coil to ref. coil)	Range:	10 km (6.21 mi)
		Dynamic Range @ 1 kHz:	58 dB
		Noise Floor:	11 mrad
		Acquisition Gauge Length:	5.1 m (16.74 ft)
		Acquisition Spatial Resolution:	5.1 m (16.74 ft)
		Acquisition Spatial Sampling Interval:	2.55 m (8.37 ft)
		Acquisition Frequency (repetition rate):	10 kHz
		Recording Length:	30 s
